# Device Therapy in Children: Current Indications

**Published:** 2008-05-01

**Authors:** N Sreeram, U Trieschmann, E de Haan

**Affiliations:** University Hospital of Cologne, Germany

**Keywords:** pediatric, device therapy, indications

## Abstract

The implantable cardioverter defibrillator has achieved increasing acceptance in paediatric cardiologic practice. Concurrent with technological advances which have made the devices more versatile, easier to implant and to program, there has been a fundamental breakthrough in our understanding of genetic and inherited arrhythmia syndromes in the last decade. This in turn has led to investigations into risk stratification, with the aim of choosing high risk candidates for timely device therapy. The second group of young patients with a risk of sudden death are those who have had a previous repair of a structural heart defect. Given that sudden arrhythmic death is the commonest cause of mortality in this population, it behoves the practising paediatric cardiologist to be aware of the current recommendations for device implantation in this population. In this manuscript, we summarise the current state of our understanding of the risk factors for sudden death, and identify possible candidates for ICD implantation.

## Introduction

It is well recognised that implantable cardioversion / defibrillation devices (ICDs), can be potentially lifesaving in children at risk of arrhythmic sudden death. In a wide variety of settings however, the appropriate selection criteria for ICD implantation at a young age remain unclear. This has resulted both in device over-utilisation in certain disease categories, and potential under-utilisation in others. We shall attempt to provide some guidelines on the appropriate indications, and the potential benefits and risks of ICD implantation in this population.

The role of ICDs may broadly be considered under two headings: devices for primary electrical disease, and devices to prevent sudden arrhythmic death in patients who have previously undergone surgical repair for congenital heart defects.

## Primary Electrical Disease

In 1996, Maron et al provided autopsy data on the causes of sudden unexpected death in 134 young athletes aged <40 years. Approximately 45% of the deaths could be attributed to potentially inherited diseases [1]. Two other studies looking at the causes of sudden death in young individuals (non-athletes) reached roughly similar conclusions [2,3]. In recent years, rapid advances in our understanding of the function of cardiac cellular ion channels, their role in generating and maintaining the normal cardiac action potential, and the arrhythmogenic effects of impaired/ altered function resulting from mutations affecting the genes which regulate channel structure and function, have all led to a surge of interest in ion channel disease, or the channelopathies. Mutations in different genes can cause a range of congenital arrhythmia syndromes. These mutations can produce diverse functional effects in utero, and clinical overlap into different disease phenotypes resulting from a single gene mutation, particularly for sodium channel gene mutations, are recognised. The clinical features of a genetic mutation affecting one of the ion channel proteins can vary not only according to genotype, but also by the specific mutation.

### The congenital long QT syndrome

The disease which has been recognised the longest, and consequently has received the most attention, is the congenital long QT syndrome. The congenital LQTS is estimated to affect approximately 1 in 5000 persons, and the first chromomal defects causing LQTS were identified in 1995 [4,5]. Eight genotypes (over 400 mutations) are known to date, seven of them being ion channel gene mutations, and one of them (LQT4) a structual anchoring protein mutation. Beta blockers are the mainstay of management, although occasionally other drugs may be tried for specific genotypes. Left cardiac sympathetic denervation (LCSD) may be tried in patients with severe clinical phenotype associated with QTc>500. Although not completely protective, in responders (those in whom the QTc decreases to <500 within 6 months of LCSD) a significant reduction in the incidence of syncope and aborted sudden death have been reported [6].

### Indications for ICD in LQTS

Class I indications for ICD in congenital LQTS include resuscitated ventricular fibrillation or aborted sudden death. Additional clinical states in which ICDs may be considered include a family history of sudden death in a first degree relative in conjunction with a QTc >500, and recurrent syncope despite appropriate pharamacologic compliance with beta-blocker therapy. Attempts at more sophisticated risk stratification based on genotype, sex and QTc have generally not been well accepted in paediatric practice, as the overlap between the different risk categories is too large. It is important however to recognise that LQT1, LQT2 and LQT3 together account for approximately 70% of all patients with LQTS, and exhibit specific genotype-phenotype relationships. Thus arrhythmic events in LQT1 are most commonly triggered by exercise or emotional upset (sympathetic overdrive); clinical events in LQT2 are most commonly triggered by sudden emotional upset (including auditory stimuli), while LQT3 related events occur predominantly at rest [7]. Appropriate steps need therefore to be taken to avoid triggering events. Exercise testing may be helpful in LQT1, to confirm non-shortening of the QT interval with exercise, as part of the risk-stratification algorithm.

Recent interest has focused on mutation-site specific differences in arrhythmic risk and in sensitivity to sympathetic stimulation in LQT1. Transmembrane domain mutations have been shown to cause a greater prolongation of various measured repolarization parameters, and to be associated with a greater clinical risk of arrhythmia when compared to C-terminal mutations [8]. Such data may in future refine the ability to define risk of sudden death in a specific patient in the future, although they are of limited value at present. Finally, specific single mutations, or compound heterozygous states can be associated with a severe clinical phenotype, emphasising the importance of genotyping whenever possible.

There are several theoretical and practical considerations when implanting an ICD in a young population, a detailed description of which is beyond the scope of this review. In general, the incidence of inappropriate shocks is high in the paediatric population, related to a variety of reasons (sinus tachycardia, supraventricular arrhythmia, nonsustained VT, T wave oversensing or electrode defects). Korte et al demonstrated in a varied population of young patients that there were 1.5 appropriate therapies versus 1.3 inappropriate ICD therapies per patient year of follow-up [9]. Electrical storms (appropriate shock → emotional distress → sympathetic overdrive → more arrhythmias → more shocks) leading to non-acceptance of the device by the patient and family have also been reported. Despite the use of various sophisticated ICD algorithms (increasing the detection time and rate, use of dual chamber systems with atrial discrimination algorithms, detection enhancements such as sudden onset, rate stability, QRS discriminators and the use of post-shock high-rate pacing) these problems have not been abolished in LQTS patients [10].

### A role for permanent pacing in LQTS?

The rationale for permanent pacing in the LQTS is that temporary pacing, regardless of pacing mode, results in a uniform decrease in QT interval. Pacing also avoids beta-blocker induced bradycardia, and protects against pause-dependent torsade de pointes [11]. Pacing alone however is not completely protective [12]. At present, permanent pacing is confined to neonates and infants with severe symptomatic bradyarrhythmia, either sinus bradycardia as seen in LQT1 or functional 2:1 AV block as occurs with homozygous LQT2 mutations or an LQT2 mutation in association with another mutation [13,14]. The majority of such patients have QTC>550, and are at risk of syncope, haemodynamic compromise and malignant ventricular arrhythmias. High pacing rates (100 to 110 beats/min) are usually required for the first 2 years of life, and pacing may be considered a bridge to ICD implantation.

### The short QT syndrome

This relatively recently recognised genetic syndrome is uncommon. The electrocardiographic hallmark is a short QT interval of <300ms. It can present at a very young age, and carries a high risk of sudden death, syncope and paroxysmal atrial arrhythmias. The majority of patients recognised to date have a positive family history of sudden death. Electrophysiologically, the SQT is associated with very short atrial and ventricular refractory periods. Three disease variants (SQT1 to SQT3) have been identified thus far, and all of them are associated with gain of function mutations of one of the cardiac ion channels, resulting in abrupt termination of repolarization [15-17]. When recognised, ICD therapy appears to be uniformly indicated, in view of the malignant phenotype. There have been limited data concerning the efficacy of quinidine in prolonging and even normalising the QT interval, and in rendering ventricular arrhythmia non-inducible [18]. At present, quinidine may be considered in addition to an ICD, to limit the number of ICD discharges.

### The Brugada syndrome

This is an inherited arrhythmogenic disorder characterised by a typical electrocardiographic pattern. It has been linked to mutations in the sodium channel (SCN5A) gene, although to date such mutations have been identified in only 20 to 30% of patients with definite or suspected Brugada syndrome. The disease is clinically heterogeneous, and has been linked to sudden cardiac death in the young, being considered responsible for approximately 20% of sudden death in patients with structurally normal hearts. The typical symptomatic patient however is the young adult male, with a reported mean age at sudden death of around 40 years. From predominantly adult studies, the risk factors for sudden death have been identified to be a spontaneously occurring type 1 electrocardiogram (particularly in males, although a spontaneous type 1 ECG appears to be less common in high-risk female patients) in association with a history of syncope or aborted sudden death [19,20]. The role of invasive electrophysiological studies in stratifying risk remains controversial; the worse outcome in the Brugada registry for patients with inducible ventricular arrhythmia may reflect a selection bias. What is generally agreed upon however is that electrophysiologic study has a good negative predictive value - the majority of asymptomatic patients who are non-inducible at EPS remain asymptomatic at follow-up [21].

Data in children are derived largely from a single multicenter study of 30 patients (age <16 years) from 13 different European centers [22]. These data confirm what is known from adult practice; the majority of symptomatic children had a spontaneously occurring type 1 ECG. There was no male preponderance in this population, and family history was unhelpful in predicting risk of arryhthmia. The majority of syncopal events occurred during rest, and fever was an important trigger of arrhythmic events (syncope or sudden death). The incidence of atrial arrhythmias was also higher than has been reported in the adult population. As might be expected from such a heterogeneous group, no standardised diagnostic or therapeutic protocols were followed. Only a limited number of patients underwent invasive electrophysiologic studies, and ICDs were implanted in five children (four of whom had had symptoms). In addition four children were treated with quinidine, and none of them (including two who had previously been symptomatic) had an arrhythmic event during a limited mean follow-up interval of 28 ± 24 months. It is not possible on the basis of this report to recommend quinidine as an alternative to an ICD in symptomatic patients.

From these and other data, it appears that drug challenge to unmask the typical type 1 electrocardiographic pattern, while helpful in establishing the diagnosis in individuals with a positive family history, does not appear to be useful in identifying patients at risk. In these patients, annual follow-up with ECG recording appears to be adequate, to monitor for the occurrence of a spontaneous type 1 ECG. In a limited series of children (own unpublished data) with a strong family history of sudden death or aborted sudden death, invasive EP studies performed in a standardised manner have not thus far been positive in the absence of prior symptoms. The indications for ICD implantation concur with those used in adult practice - a spontaneous type 1 ECG in association with symptoms. An important caveat is that in patients in whom the diagnosis has been established, febrile episodes have to be treated in a timely fashion, to avoid fever-related arrhythmic events.

### Catecholaminergic polymorphic ventricular tachycardia (CPVT)

This syndrome has its onset at a young age. It is characterised by ventricular arrhythmias occurring during exercise, when the heart rate typically exceeds a threshold of between 120 and 130 beats /min. Familial forms account for approximately 30 to 50% of CPVT, with both autosomal dominant and recessive forms (ryanodine RyR2 receptor gene and calsequestrin 2 gene defects) being recognised. This phenotype is associated with a high mortality, with 30 to 35% of patients with the clinical disease dying before the age of 30 years. In a recent autopsy study of young patients dying suddenly, Tester et al identified RyR2 mutations in seven of 49 individuals (14%) postmortem, suggesting that CPVT may be underdiagnosed in life [23].

Beta blockers, at a maximally tolerated dose, form the mainstay of therapy. In selected patients with symptoms despite maximal beta-blocker therapy, left cardiac sympathetic denervation may be helpful in controlling symptoms [24]. The role of ICDs is controversial. Small studies have confirmed the efficacy of ICDs in preventing sudden death [25]. As for the LQTS, long detection times and post-shock pacing / rate-smoothing algorithms will be required to prevent excessive shocks and electrical storms.

### Arrhythmogenic right ventricular dysplasia (ARVD)

ARVD is a primary cardiomyopathy, which is often familial, and is characterised by fibrofatty replacement of the right ventricular myocardium, which in turn can cause sudden arrhythmic death in young people. The diagnosis can be difficult, and often requires a combination of family history, electrocardiographic abnormalities (depolarisation/repolarisation and conduction defects) in association with right ventricular arrhythmias, global or regional right ventricular dysfunction and specific histologic changes in the right ventricle.

The indications for ICD implantation in ARVD have been well established from various studies in Italy and the mediterranean region, where the disease is most commonly encountered. These include a history of aborted sudden death, syncope, sustained ventricular tachycardia (with or without haemodynamic compromise), and non-sustained ventricular tachycardia in association with a positive family history for sudden death at a young age (<35 years). Slightly less well accepted as an indication for ICD is the combination of inducible sustained ventricular tachycardia in association with a positive family history of sudden death. When an ICD was implanted for one of the above indications, Corrado et al have demonstrated a 15% rate of appropriate interventions per year of follow-up [26]. The incidence of appropriate ICD discharges appears to be roughly equal at upto 48 months of follow-up, for each of the above indications, and has been shown to be associated with a clear survival benefit [26]. Concerns remain about lead complications at follow-up (undersensing, pacing failure, requirement for additional patches and high energy devices), due to the progressive nature of the right ventricular dysplasia. Though right ventricular perforation appears to be uncommon, it is not unusual that more right ventricular lead positions need to be tested, and that the final R wave amplitude is significantly lower compared to normal hearts [26,27].

### Hypertrophic Cardiomyopathy

Hypertrophic cardiomyopathy is a progressive primary myocardial disease, whose histological hallmarks are myofibrillar disarray and subendocardial ischema/necrosis related to the degree of hypertrophy. Several genetic variants of the disease have been recognised. Secondary prevention indications for ICD implantation include aborted sudden death and documented ventricular tachyarrhythmias with syncope/presyncope [28]. Much interest has been generated in the area of establishing primary prevention indications for ICD implantation in this patient population. At present, some of these criteria include a malignant genotype, sudden cardiac death in family members, unexplained syncope, abnormal blood pressure response to exercise, severe left ventricular hypertrophy with a free wall thickness of the left ventricle in excess of 30mm, and induced ventricular tachyarrhythmia or atrial fibrillation [29]. Follow-up data of ICD implantation for secondary prevention show an appropriate annual discharge rate of 10-11%. When ICDs have been implanted for primary prevention of sudden death, and when two or more of the risk factors enumerated above had been present (the European approach), there is a 5% per annum incidence of appropriate discharges [29]. More recently, in selected high risk patients, the presence of a single risk factor has been shown to justify justify ICD implantation [30]. In a multi-center study, Maron et al. demonstrated that appropriate ICD discharges occurred in 35% (18/51 patients, in whom monitored risk factors included family history for sudden death, severe left ventricular hypertrophy, nonsustained ventricular tachycardia on Holter recordings, unexplained syncope) of patients who had undergone ICD implantation for a single risk factor. The likelihood of appropriate discharges was also similar for patients undergoing primary prevention ICD implant for 1, 2 or 3 or more risk markers [30]. It is likely therefore that ICD use even with one risk marker will gain more widespread acceptance. The interval to first appropriate discharge however was upto 10 years, with a 27% probability of first appropriate discharge occurring five years or later following ICD implant.

There are little data on ICD implantation in children. In a recent publication, Kaski et al reported on follow-up data after ICD implantation in 22 patients with hypertrophic cardiomyopathy aged <16 years [31]. Indications for implant included occurrence of a pervious cardiac arrest, or the presence of two or more of the previously enumerated risk factors for sudden death. Over a short follow-up interval (mean 1.7 years, range 1-2.3 years), the annual appropriate ICD discharge.rate was 71.4% for the secondary prevention group, versus 4.1% for the primary prevention group. The 5 year shock-free survival rates for secondary prevention versus primary prevention were 40% versus 93.3%. Bearing in mind that the risk of sudden death in HCM is lifelong, and that intervals to appropriate discharges may be >10 years in individual patients, these data do not necessraily detract from the life-saving role of ICDs in this patient population.

The left ventricular outflow tract gradient at rest, in the absence of other risk factors, does not appear to predict the risk of sudden death [32]. Further studies need to be undertaken on how best to manage patients with a combination of severe outflow obstruction and one or more risk factors for sudden death (ICD implantation versus gradient reduction or a combination of the two). In addition, transcatheter transcoronary alcohol ablation of septal hypertrophy also does not appear to influence the rate of appropriate ICD discharges [33].

## ICDs to Prevent Sudden Death Following Surgical Repair of Structural Heart Defects

Population-based studies have established that the cumulative risk of sudden death following surgical repair of congenital structural heart defects is low [34]. Postoperative arrhythmias however are relatively common, and can be an important clinical burden for the patient. Patients at risk of sudden death usually have associated chronic pressure or volume overload of the ventricle. In this setting, myocardial stretch, ischemia, fibrosis, electromechanical coupling and ventriculo-ventricular interaction may all be causative factors for arrhythmia. The observation that the majority of children and young adults with structural heart disease are likely to have a ventricular arrhythmia as their presenting arrhythmia during cardiac arrest has focused attention on identifying patients at risk of postoperative VT. The four categories of lesions that are commonly considered to be associated with a risk of sudden death following surgical repair include tetralogy of Fallot, D-transposition of the great arteries following a Mustard or Senning repair, congenital aortic stenosis and associated left ventricular outflow tract obstruction variants, and palliated univentricular hearts [35].

As a general rule, aborted sudden death and recurrent haemodynamically unstable polymorphic ventricular tachycardia with synope/presyncope would constitute definitive indications for ICD implantation. Invasive electrophysiological studies have been performed in several settings to attempt to identify patients most likely to benefit from an ICD. Alexander et al studied 130 patients (mean age 18.7 ± 10.6 years). The patients fell into one of the four disease categories enumerated above. Indications for the EP study included symptoms (palpitations, syncope), and documented sustained or non-sustained VT. Approximately 14% of patients had experienced a near-miss sudden cardiac death event. In this carefully selected population, a positive V-STIM study had a high sensitivity (87%), in predicting future mortality. A false negative V-STIM outcome however was obtained in upto 33% of the study population. The conclusions drawn from the study were that a positive V-STIM identifies a high risk subset of patients, while a negative study does not exclude future life-threatening arrhythmic events [36].

### Tetralogy of Fallot

Sudden cardiac death is the most important cause of mortality after repair of tetralogy of Fallot, with an annual mortality rate of 0.3% [35]. Risk factors for sudden death include older age at repair, a longer duration of follow-up, presence of a right ventricular outflow tract surgical patch, high grade ventricular ectopy and early transient heart block [37-39]. Several electrocardiographic markers of sudden death have been identified, including QRS duration (>180ms, 100% sensitivity), annual change of QRS duration (in msec/year), and increased QT and JT dispersion [40,41]. The haemodynamic risk factors associated with ventricular arrhythmia include right ventricular pressure and volume overload, and rather surprisingly (and probably underappreciated), left ventricular dysfunction from ventriculo-ventricular interaction [38,42,43]. The combination of QRS duration and the presence or absence of left ventricular dysfunction appeared to have the best positive and negative predictive value for sudden death [44].

#### EP study in tetralogy of Fallot

The largest series of patients undergoing EP study following repair of tetralogy of Fallot was reported on by Khairy et al [45]. They studied 252 patients (age 16 ± 12.3 years). Only 25% of this patient cohort had had a documented VT or aborted sudden death, and 36% of this population were undergoing EP study as part of routine screening. The positive predictive value of V-STIM in this rather unselected population was 55%, while the negative predictive valuewas 91%. The authors reasonably concluded that induction of monomorphic or polymorphic ventricular tachycardia at EPS was predictive of future arrhythmic events. It is important to recognise however that a single negative EP study does not provide the patient with a lifelong clean bill of health. Issues that need to be studied in the future include recognition of the objective criteria for repeating an EP study, even when the initial one was negative.

#### Surgical repair of residual haemodynamic sequelae

The role of surgical correction of residual haemodynamic sequelae to prevent or cure ventricular arrhythmia has also been studied. Therrien et al reported on a series of 70 patients who underwent pulmonary valve replacement for chronic pulmonary insufficiency following tetralogy repair. Intraoperative cryoablation was performed in 9 patients who had had previous ventricular arrhythmias. At follow-up, there was a marked decrease in the incidence of ventricular arrhythmias (from 22% to 9%), and none of the patients who had received cryoablation had had a recurrence of tachyarrhythmia at a mean follow-up interval of 4.7 years. This beneficial effect was however bought at a peri-operative mortality rate of 4% [46].

#### Ablation/ anti-arrhythmic medications

Macro-reentrant tachycardia circuits can be successfully ablated (catheter ablation or surgery), and a wide variety of anti-arrhythmic medications has been used to suppress ventricular ectopy. The role of either of these therapeutic approaches in preventing sudden death is unknown.

#### ICDs in tetralogy of Fallot

Two recent studies have shed light on the role of ICDs in patients with repaired tetralogy. The largest series from North America included 121 patients with a mean age of 32 years, and a mean follow-up interval of 3.7 years [47]. Sixty eight patients (56%) had received an ICD for primary prevention, while the remainder (44%) received the ICD for secondary prevention. The freedom from appropriate ICD discharges at 1, 2 and 5 years post-ICD implant for primary prevention were 85%, 80% and 67% respectively, compared with 79%, 66% and 54% in the secondary prevention population. The independent risk factors for appropriate ICD discharge in the secondary prevention population included (not unexpectedly) syncope/presyncope, inducible sustained polymorphic ventricular tachycardia, and the use of a class 1A or 1C antiarrhythmic drug at hospital discharge. In the primary prevention group, the most important independent predictor for appropriate ICD discharge was a higher left ventricular end-diastolic pressure. This study emphasises the need to distinguish between appropriate ICD discharges versus potentially life-saving discharges in the future, when considering ICD implantation for primary prevention. Approximately 33% of patients without previously documented arrhythmias had had an appropriate ICD discharge within 5 years of device implantation, and it is not clear in how many of them the device may have not strictly been indicated. This issue was highlighted by a second recent European study [48]. In this study, 40/64 patients with congenital heart disease undergoing ICD implantation (all aged >18 years) had a diagnosis of repaired tetralogy. The index event for ICD implantation included cardiac arrest (n=6), spontaneous sustained VT (n=18, predominantly monomorphic VT at an average cycle length of approximately 300ms and which was clinically well tolerated), syncope (n=11), palpitations (n=1), and various other reasons (n=4). At follow-up, 160/204 shocks were inappropriate, and 49/64 patients in this cohort experienced an inappropriate shock. There were no predictors of inappropriate shocks, except the diagnosis of tetralogy of Fallot. A total of 3 possibly life-saving shocks (for polymorphic VT or ventricular fibrillation) were documented in the entire tetralogy subgroup, and it was unclear whether all these shocks had occurred in the same patient. These data reemphasise the need for stricter criteria when implanting an ICD, and on the need to recognise and treat stable and well-tolerated VT by other means.

### Sudden death after the Mustard or Senning procedure for transposition of the great arteries

The reported late mortality following atrial repair for D-transposition of the great arteries ranges between 3 and 16%. Predictors of late mortality include advanced NYHA functional class, pulmonary hypertension and systemic ventricular dysfunction [49-51]. Sudden death has been reported to be the commonest cause of death in several studies [49-51]. Kammeraad et al studied the predictors of sudden death in this patient population [52]. They identified 47 patients with sudden cardiac death or aborted sudden death. Each patient was matched with two controls of a similar age, who had undergone the same operation at the same center, within a similar time-frame. The mean age at the sudden death event was 12.3 years. Sinus node disease was present in 59%, atrial flutter in 42%, a combination of SND and AF in 30%, and permanent pacemakers in 17%. The documented rhythm (when available) at the SD event was ventricular tachycardia or fibrillation in the majority of patients. The presence of symptoms (of either heart failure or arrhythmia) and documented atrial flutter were strong predictors of sudden cardiac death.

#### ICDs following atrial repair of TGA

Secondary prevention indications for ICD implantation are reasonably well agreed upon, and include aborted sudden cardiac death and sustained ventricular arrhythmias. The role of atrial arrhythmias in causing sudden death is unknown. Khairy et al recently reported on the largest cohort of ICD recipients following atrial repair of TGA [53]. Appropriate ICD discharges at follow-up were almost entirely confined to secondary prevention patients. Independent predictors of appropriate ICD discharges included moderate or severe systemic AV valve regurgitation and the absence of beta blocker therapy. In conjunction with the findings of the Kammeraad study where documented atrial arrhythmias predicted sudden death, it may be speculated that beta blockers may have had a role in suppressing atrial arrhythmias in this patient population. Interesting issues which require further consideration and study include the following: (1). Should patients with documented atrial flutter routinely undergo a V-STIM study? (2). At the time of a routine pacemaker revision, should a V-STIM study be performed, to make the decision to upgrade to an ICD?

## Conclusions

The optimal treatment for preventing sudden death in post-surgical patients with congenital heart disease is likely to involve a hybrid approach, using a combination of haemodynamic repair, surgical or catheter ablation of macro-reentrant ventricular tachyarrhythmias, catheter ablation or pharmacologic therapy for atrial arrhythmias, and ICD implantation. Risk assessment in ths patient population remains challenging due to the heterogeneity of structural heart diseases, the unpredictable progression of adverse haemodynamic sequelae and their effect on ventricular function, the unpredictable timing of clinical events, and not least the low annual mortality rates.
